# Penetrating head trauma: 03 rare cases and literature review

**DOI:** 10.11604/pamj.2017.28.305.10376

**Published:** 2017-12-11

**Authors:** Youssef Fahde, Mehdi Laghmari, Mohamed Skoumi

**Affiliations:** 1 Department of Neurosurgery, Arrazi Unit, Mohammed VI Hospital, Cadi Ayad University, Marrakech, Morocco

**Keywords:** Penetrating head trauma, CT scan, craniectomy, infection

## Abstract

Penetrating head trauma (PHT) include all open head injuries with foreign object in the brain. Although less common than closed head trauma, penetrating head trauma carry a worse prognosis. We received three unusual cases of penetrating head injuries whose prognosis was different according to clinical presentation and initial management of the patient. Treatment of penetrating head trauma aims at controlling bleeding, controlling intracranial pressure and preventing infections. Despite the efforts made by national authorities as well as the adequate management in hospitals, penetrating head injuries are still frequent with significant mortality and morbidity.

## Introduction

Penetrating head trauma (PHT) includes all traumatic brain injuries which are not the result of a blunt mechanism [1]. Although less prevalent than closed head trauma, PHT carries a worse prognosis [2]. PHT is still common in our country because of road traffic accidents, physical assault by non-missile, low-velocity objects. It represents a rare pathology among civilians, with better outcome because of more localized injury [2,3] and is usually caused by violence, accidents, or even suicide attempts [4, 5]. Many trauma centers and emergency services all over the world still receive large numbers of traumas patients as a result of the public road accidents and assaults, despite all the judicial efforts of law organization, which has therefore made the formulation of standard surgical and management algorithms for PHT patients to become capital [1], leading to different health associations and organizations collaborating in order to establish evidence based guidelines. In 1995 the American Association of Neurological Surgeons and the Brain Trauma Foundation came up with guidelines for the management of severe head trauma, which is periodically revised and last updated as third edition in 2007 [6,7]. In the spring of 1998, the international brain injuries association, the American association of neurological surgeons and the congress of neurological surgeons come to complement these guidelines by addressing the management of penetrating cranio-cerebral traumas (such as gun shots, stab wounds…). The result of this work was published in 2001, which attempted to standardize the medical and surgical management of these traumas [8]. In this article the authors expose their own experience with rare and impressive illustrative cases.

## Patient and observation

### Case 1

**Figure 1:**
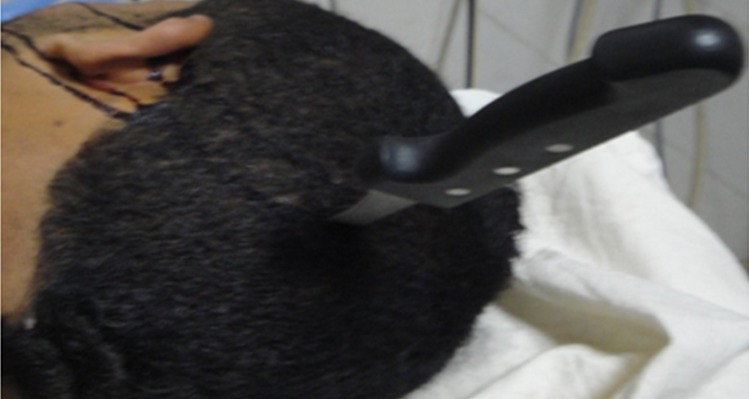
Patient with a knife inside the left occipital area of the skull

**Figure 2:**
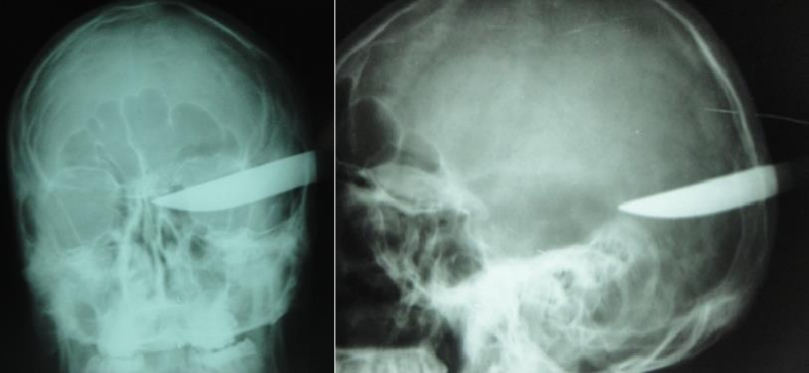
Skull X-ray shows the knife penetrating the skull

**Figure 3:**
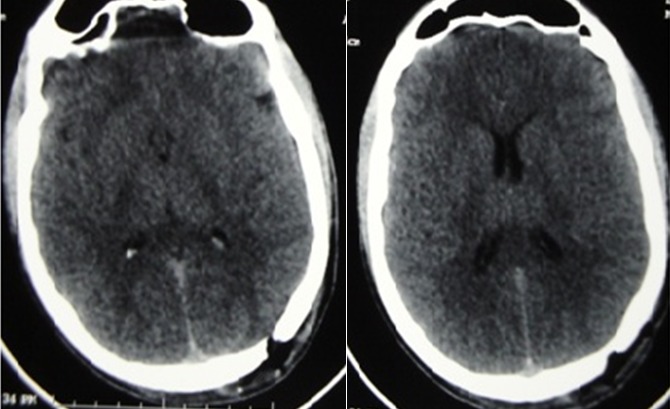
Scanner of control for the patient in figure one showing nearly complete consolidation

Eighteen years old boy presented to the emergency department after an assault by penetrating knife in his left side of skull (Figure 1). Patient was conscience and could mobilize superior and inferior limbs. He complained of headache and repeated vomiting. Patient was vitally stable. Skull X-ray and CT scan showed 20cm knife planted 5cm deep into the left occipital lobe (Figure 2). Surgical treatment consisted to a small craniectomy around the knife, Cleaning and debridement of necrotic tissue and repair of anatomical structures, with dura mater plasty after safe hemostasis by bipolar forceps and application of oxidized regenerated cellulose. Combined antibiotics ceftriaxone and metronidazole were administered as well as an anticonvulsive, antitetanic vaccine, and analgesics. The patient was discharged from hospital after 03 days without postoperative complication. CT scan of control was excellent (Figure 3).

### Case 2

**Figure 4:**
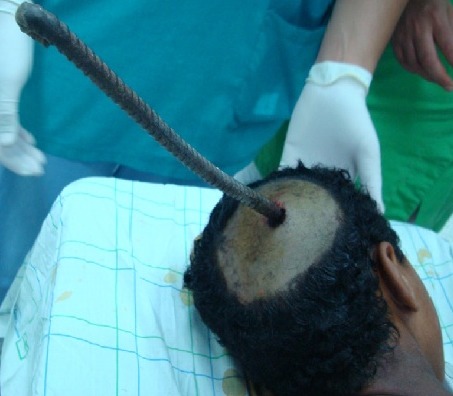
Patient with a metallic rode inside the right side of the head

Fourteen years old boy presented to emergency department with a penetrating metallic rod inside the right occipital side of the head (Figure 4). Patient was confused with repeated vomiting. We noted a mild right hemi paresis. Radiological examination confirmed the diagnosis. The metallic object was removed by a craniectomy. Necrotic structures were resected and washed abundantly. A dura mater plasty was crafted after hemostasis. Medical treatment consisted of antibiotics, anticonvulsive, antitetanic vaccine and analgesics. He underwent 10 sessions of physiotherapy for the right hemiparesis and after one month of follow-up he became normal.

### Case 3

**Figure 5:**
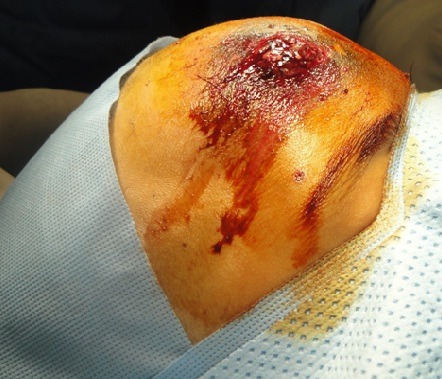
Patient with discharge of cerebral tissue from the forehead of ballistic injury

**Figure 6:**
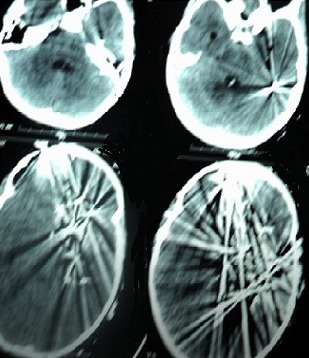
Scanner of the same patient in figure five showing the present of multiple metallic fragments inside the brain

**Figure 7:**
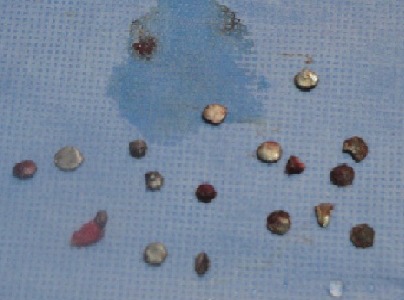
Image of the eighteen fragments which we can remove from the patient in figure five

Three years old girl victim of penetrating head injury caused accidentally by explosion of her father’s fire arm. The patient was admitted after a long distance and bad conditions of transfer. The patient presented in a state of coma with glasgow coma scale at 4/15, decerebration, discharge of brain tissue from a wound in the forehead (Figure 5). Patient was vitally unstable. Scanner confirmed penetration of large numbers of metallic fragment inside the frontal lobe (Figure 6). The surgical treatment consisted of large frontal bone flab. Eighteen fragments from extra cerebral and intra cerebral regions were removed (Figure 7). Meticulous cleaning of the wound and plasty of dura mater. Although put under medical treatment in the intensive care unit the patient died after six hours because of severity of the initial lesion.

## Discussion

Penetrating head injuries are mostly caused by ballistic high velocity objects, which results in more complex injuries and high mortality. PHT caused by non-missile, low-velocity objects represents a rare pathology among civilians, with better outcome because of more localized primary injury, and is usually caused by violence, accidents, or even suicide attempts [3]. Optimum management of PBI requires a good understanding of the mechanism of injury and its pathophysiology [9]. As most of the PHTs are caused by missiles or projectiles, an understanding of ballistics is imperative. PBI is expected to be much more severe in case of a close range fire arm injury as maximum amount of initial kinetic energy is transferred to the brain tissue and blast effects. It is not clear which technique, craniotomy, or craniectomy, is best to achieve the most optimal results [10].

A recent large study of military patients suffering from severe PHT reported optimum outcomes with early decompressive craniectomy. The risk of local wound infections, meningitis, ventriculitis, or cerebral abscess is particularly high among PHT patients because of the presence of contaminated foreign objects like skin, hair and bone fragments. The risk of posttraumatic epilepsy after PHT is high probably due to direct traumatic injury to the cerebral cortex with subsequent cerebral scarring [1]. Penetrating brain injuries are common in our region, the authors present three unusual cases to illustrate the current situation and prognosis. Treatment of PHT aims at immediately saving life through control of persistent bleeding and intracranial hypertension, prevention of infection through debridement of all contaminated and necrotic tissues, antitetanic vaccine and prophylactic antibiotics. Preservation of as much nervous tissue as possible and restoration of anatomic structures through accurate closure of the dura and scalp are mandatory.

Penetrating head injuries have been extensively reported in the literature since the first publication of a stabbing trauma through the cranium in 1848 [11]. The major consequences of low velocity stab wounds to the brain are hemorrhage and infection. Head X-ray and CT should be done if there is a suspicion of an intracranial penetrating injury. MRI can be dangerous in the case of retained ferromagnetic objects due to possible movement of the object in response to the magnetic torque [12]. Wood splinters and bone fragments are the most common cause of intracerebral infection, which did not happen in our cases. Abscess typically develops within three to five weeks after injury with Staphylococcus Aureus being the most common organism. Antibiotic therapy typically begins with a combination of metronidazol for anaerobes and a late generation cephalosporin [13]. Less frequent complications of penetrating brain injuries are cerebrospinal fistula and neuroendocrine dysfunction [13].

## Conclusion

This brief report serves to highlight that penetrating head injury can occur without neurological deficit. It is possible to remove objects penetrated in the brain with a minimally invasive approach, when the potentially associated vascular injuries have been ruled out and appropriate antibiotic coverage is given.

## Competing interests

The authors declare no competing interests.
